# Editorial: Insights in: evolutionary psychology 2022

**DOI:** 10.3389/fpsyg.2024.1458388

**Published:** 2024-08-05

**Authors:** Karlijn Massar, Mark Collard, Peter K. Jonason

**Affiliations:** ^1^Department of Work and Social Psychology, Maastricht University, Maastricht, Netherlands; ^2^Department of Archaeology, Simon Fraser University, Burnaby, BC, Canada; ^3^Department of Psychology, University of Padua, Padua, Italy; ^4^Department of General Psychology (DPG), Institute of Psychology, University of Cardinal Stefan Wyszynsky, Warsaw, Poland

**Keywords:** evolutionary psychology, insights 2022, attachment system, facial attractiveness, hormones, personality traits

The aim of the current Research Topic was to highlight current advancements in evolutionary psychological research. The contributions highlight the diversity of theoretical and methodological approaches utilized by scholars in the field, and together, they shed more light on the evolutionary origins of human cognition, emotion, and behavior.

In their contribution, Kotov and Corpuz report on a relatively understudied area within the research on the influence of hormones on human males' psychology. The interaction between testosterone and cortisol (i.e., the Dual Hormone Hypothesis) has been established in regulating mate traits associated with dominance. It is less well-known how these hormones influence traits that are ostensibly opposed to status seeking (i.e., depressive symptoms). These authors test the relationships between the DHH and depressive symptoms in first-time fathers, thereby furthering the knowledge on the neuroendocrinology of postpartum depression among fathers.

Gagliardi explores the adaptive function(s) of the human attachment system, hypothesizing that it is a motivational and data system infants and children use to gather information from caregivers, and to optimize future interactions. The author further proposes that whereas somaticity modulates affiliation tendencies, when there is a mismatch between the current and learning environments, it can potentially increase vulnerability to social anxiety and eating disorders. This contribution has relevant implications for evolutionary psychiatry and clinical psychology by integrating multiple levels of explanation for psychopathology, thereby informing the design of new psychological treatments.

Utilizing genome-wide SNP data (713,014 SNPs) of participants from the Wisconsin Longitudinal Study, Fieder and Huber test the assumption that genetic heterozygosity, particularly within the major histocompatibility complex (MHC), is positively associated with facial attractiveness. Specifically, heterozygosity at certain loci may signal a preferable genetic makeup (e.g., pathogen resistance), potentially influencing mate choice. Although the authors do find an association between increased homozygosity and attractiveness, these effects were small and not robust against multiple testing. Thus, although heterozygosity, and thus vulnerability to pathogens, may be detected by attractiveness to a small extent, the authors emphasize the need for bigger and cross-cultural samples.

Lastly, the contribution by Gutiérrez et al. deepens life history theory, by analyzing the associations between reproductive traits, distinct mating strategies, and personality traits. The authors identify two pathways to a fast life history strategy: One through fewer but longer lasting relationships (fast-restricted), and another through more but shorter relationships (fast-unrestricted). Thus, contrary to common assumptions, these results indicate that reproductive success is linked more to relationship duration than to the number of mates. The authors emphasize that human reproductive behavior is multifaceted, and that a nuanced understanding of reproductive strategies in humans should include attention to ecological conditions, culture, and personality traits.

Together, the contributions in this Research Topic highlight several common themes. First, they all aim to deepen our understanding of complex phenomena within human psychology and behavior, whether it's the influence of hormones on depressive symptoms, the adaptive function of the attachment system, genetic influences on facial attractiveness, or the diversity of reproductive strategies and their association with personality traits. Second, they challenge traditional assumptions and theories, by revealing nuanced relationships and interactions between various factors. Finally, they emphasize the importance of considering multiple levels of explanation, including genetic, hormonal, psychological, and environmental factors, to fully comprehend human psychology and its evolutionary roots.

## Author contributions

KM: Writing – review & editing, Writing – original draft. MC: Writing – review & editing, Writing – original draft. PJ: Writing – review & editing, Writing – original draft.

